# Dopamine Enhances Expectation of Pleasure in Humans

**DOI:** 10.1016/j.cub.2009.10.025

**Published:** 2009-12-29

**Authors:** Tali Sharot, Tamara Shiner, Annemarie C. Brown, Judy Fan, Raymond J. Dolan

**Affiliations:** 1 Wellcome Trust Centre for Neuroimaging, Institute of Neurology, University College London, London WC1N 3BG, UK; 2 Undergraduate Program, Connecticut College, New London, CT 06320, USA; 3 Department of Psychology, Harvard University, Cambridge, MA 02138, USA

**Keywords:** SYSNEURO

## Abstract

Human action is strongly influenced by expectations of pleasure. Making decisions, ranging from which products to buy to which job offer to accept, requires an estimation of how good (or bad) the likely outcomes will make us feel [1]. Yet, little is known about the biological basis of subjective estimations of future hedonic reactions. Here, we show that administration of a drug that enhances dopaminergic function (dihydroxy-L-phenylalanine; L-DOPA) during the imaginative construction of positive future life events subsequently enhances estimates of the hedonic pleasure to be derived from these same events. These findings provide the first direct evidence for the role of dopamine in the modulation of subjective hedonic expectations in humans.

## Results and Discussion

An unresolved question is whether neuromodulatory systems implicated in value-based decision making, in particular dopamine (DA), impact on the generation of subjective estimations of future hedonic reactions. Dopamine is a key neuromodulator in reward learning and reward-seeking behavior in nonhuman animals [2–4]. In humans, drugs enhancing dopaminergic function (e.g., dihydroxy-L-phenylalanine; L-DOPA) have been shown to augment a striatal signal that expresses reward prediction errors during instrumental learning, thereby increasing the likelihood of choosing stimuli associated with greater monetary gains [5]. When it comes to making more complex, real-life choices, humans are endowed with an ability to mentally simulate possible future scenarios that helps us predict the likely emotional outcome of these events [6]. We have recently shown that during imagination of future events, activity in the heavily dopaminergic innervated striatum tracks subjects' estimates of the expected pleasure to be derived from those events [7]. Given this set of findings, we reasoned that if dopamine modulates reward prediction, then its enhancement during imagination of future events should impact on subjective estimations of future pleasure to be derived from those events.

**Figure 1:**
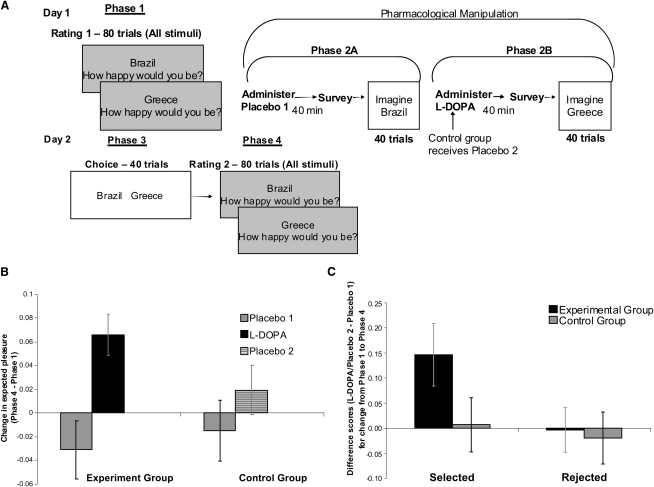
Experimental Task and Results (A) On day 1, participants rated stimuli before pharmacological manipulation (phase 1). They then completed an imagination task under placebo 1 (phase 2A) or L-DOPA (phase 2B; placebo 2 for order control group). On day 2, participants returned to the laboratory and completed a decision-making task (phase 3) and a final rating task (phase 4). For full details, see Supplemental Experimental Procedures. (B) Change in mean-corrected ratings from phase 1 to phase 4. Ratings of estimated happiness increased only for stimuli previously imagined under L-DOPA. (C) Difference scores (placebo 1 − L-DOPA; placebo 1 − placebo 2) for the change in mean-corrected ratings from phase 1 to phase 4. Ratings of estimated happiness increased from phase 1 to phase 4 for stimuli imagined under L-DOPA relative to placebo 1 only for selected stimuli. As expected, there was no difference in rating change for stimuli imagined under placebo 1 relative to placebo 2 in the control group for either selected stimuli or rejected stimuli. Error bars in (B) and (C) represent standard error of the mean. ^∗^p < 0.05.

To test this, we measured people's estimated pleasure of future events both before and after imagining those events under the influence of L-DOPA. In addition, we introduced a decision-making task to examine how this process interacts with choice. We presented 61 healthy volunteers with 80 different vacation destinations (e.g., Greece, Thailand) and asked them to rate their expectations of happiness if they were to vacation at each of these locations (phase 1, rating 1; see Figure 1A). We then administered placebo 1 and asked the participants 40 min later to complete a subjective state questionnaire (e.g., level of alertness, calm, interest). Next, we re-presented half of the destinations and instructed participants to imagine themselves spending next year's vacation at those locations (Phase 2A, imagination under placebo 1). We then administered L-DOPA (100 mg) to 29 participants (“experimental group,” randomly assigned) and a second placebo pill to 32 participants (“order control group” controlling for possible order confound, because L-DOPA had to be administered after placebo in the experimental group because of its half-life, which results in it requiring upwards of 5 hr for elimination). Forty minutes later, all participants completed the subjective state questionnaire again. The other half of the stimulus set was then presented with the same instruction as per phase 2A (phase 2B, imagination under L-DOPA or placebo 2). Participants then left the laboratory and returned 24 hr later.

On day 2 (by which time L-DOPA had been fully metabolized and eliminated), participants were presented with 40 pairs of destinations to which they had given equal ratings in phase 1. On each trial, they had to choose which of the two destinations they would rather vacation at (phase 3, choice). Note again that both destinations had been imagined the previous day, one under placebo 1 and the other under L-DOPA (or placebo 2 for the control group). Finally, all stimuli were rated again (phase 4, final rating).

To test whether administration of L-DOPA changed subjective estimations of future pleasure, we calculated for each stimulus the difference in mean-corrected hedonic ratings from pre- (phase 1) to post- (phase 4) pharmacological manipulation (see note at bottom of Table S1 available online for more information). Note that an analysis of raw scores (Supplemental Results) revealed the same findings as the analysis of mean-corrected scores reported below. A repeated-measures analysis of variance (ANOVA) (group [experiment/order control] × condition [placebo 1/L-DOPA or placebo 2] × choice [selected/rejected]) revealed an interaction between group and condition (p < 0.05). This interaction was due to an increase in ratings for stimuli imagined under L-DOPA compared to placebo 1 in the experiment group (p < 0.025; Figure 1B), with no difference for stimuli imagined under placebo 1 and placebo 2 in the control group (p > 0.8; Figure 1B). This result suggests that enhanced dopaminergic function during imagination subsequently increases estimations of future hedonic reaction (see Supplemental Results for additional ANOVA results). Importantly, dopamine did not increase feelings of happiness (see Table S1) but instead enhanced a prediction of pleasure associated with a future event.

There was a significant correlation between the overall increase in ratings for stimuli imagined under L-DOPA relative to placebo 1 and the probability of choosing stimuli imagined under L-DOPA (r = 0.5, p < 0.005). No parallel correlation was found in the order control group (p > 0.7). Furthermore, participants whose average ratings of predicted pleasure were enhanced by L-DOPA relative to placebo were also more likely to choose stimuli imagined under L-DOPA than stimuli imagined under placebo (p < 0.0001).

To further examine the relationship between rating change, choice, and pharmacological manipulation, we focused separately on the scores of selected stimuli and rejected stimuli. A two-way mixed ANOVA on scores of selected stimuli (group × condition) confirmed a significant interaction (p < 0.05). This interaction was driven by an increase in expected hedonic outcome for selected stimuli previously imagined under L-DOPA relative to placebo 1 in the experimental group (p < 0.005; effect seen in 79% of the participants), with no difference for selected stimuli imagined under placebo 1 versus placebo 2 in the control group (p > 0.9; see Figure 1C). For rejected stimuli, there was no group × condition interaction (Figure 1C). These results suggest that when estimated hedonic outcomes were enhanced by L-DOPA, those stimuli were more likely to be selected.

Previous studies have implicated the dopamine-innervated striatum in signaling expectations of pleasure during imagination of future life events [7]. The current findings provide the first evidence indicating a regulatory role for dopamine in generating such subjective hedonic expectations in humans. Note that these results should not be taken to imply that dopamine enhances the hedonic impact of reward per se. Instead, the findings indicate that dopamine modulates processes related to predictions of likely future pleasure in a manner reminiscent of its role in reward learning.

Imagination can be construed as conforming to a learning process that enables people to estimate the emotional reaction associated with a novel event [6, 7]. One possibility is that by affecting learning mechanisms, L-DOPA may strengthen this process, by affecting the association between the stimuli (e.g., “Greece”) and the brief hedonic reaction generated during imagination of the pleasurable event and/or by directly affecting the signal that mediates predictive information during an act of imagination. This latter possibility complements previous findings including data showing that a signal in the striatum tracks expected pleasure during imagination [7], as well as a growing body of literature suggesting a role for dopamine in reward learning [2–5, 8–11]. It has also been suggested that dopamine enhances incentive salience [12, 13], where according to this view the administration of L-DOPA might be construed as enhancing the incentive salience attributed to imagined stimuli. The current study cannot distinguish between these two possibilities. An enhancement of dopaminergic function could in principle directly modulate incentive salience or alternatively enhance the predicted hedonic utility associated with the vacation destinations, which in turn could boost incentive salience.

An alternative interpretation is that enhancing dopaminergic function during imagination increases the experience of pleasure during simulation of a future event by directly altering its hedonic impact. The ensuing hedonic reaction during this simulation could act as a proxy for future hedonic reactions, enhancing the predicted pleasure associated with the event. This explanation is problematic for a number of reasons. First, the notion that dopamine acts as a pleasure neurotransmitter has been discounted by a large body of recent evidence (see [12, 13] for review). Second, in the present study, we did not observe an influence of dopamine on any measure of hedonic reaction recorded during the pharmacological manipulation stage (see results for phases 2A and 2B and Table S1). However, it is possible that the effects of dopamine on valuation are not limited to future events and that enhanced dopaminergic function during imagination modulates the present value of a stimulus, an idea best tested with stimuli that can be directly experienced or consumed. A final possible mechanism is that the effects we observed reflect an L-DOPA strengthening, via hippocampus-dependent loops, of a memory trace of pleasant associations made during the imagination stage.

Understanding how hedonic expectations are formed is critical both for understanding human action, which is largely driven by estimations of future pleasure and pain [1, 6, 7], and for understanding how pleasure expectation can go awry in a multitude of neuropsychiatric disorders that implicate dopamine, such as drug addiction [14–17]. The current study highlights the neurobiological basis of this key aspect of human behavior, providing direct evidence of a critical role for dopamine in modulating the subjective pleasure expected to be derived from future life events.

## Experimental Procedures

### Participants

Participants were recruited through posted advertisements and assigned randomly to either the experimental group (16 males, 13 females, age range 21–31) or the order control group (15 males, 17 females, age range 20–34), blind to condition. Participants completed a screening form for significant medical conditions, gave informed consent, and were paid for their participation.

### Experimental Design and Task

#### Day One

*Phase 1, Rating 1.* Phase 1 consisted of 80 trials. On each trial, a name of a vacation destination appeared on screen for 2 s. The participants rated how happy they estimated they would be if they were to vacation at that location (from 1 = unhappy to 6 = extremely happy) by using the computer keypad. A fixation cross was then presented for 3 s. Before the beginning of this phase, participants completed four practice trials.

*Pair Construction.* Stimuli were paired via a MATLAB program as implemented previously [7]. Approximately 80% of trials included two options that were rated the same in phase 1, and only data from stimuli derived from these pairs were subsequently used in data analysis. The remaining pairs (approximately 20%) included two options rated differently in phase 1. Each stimulus appeared in only one pair. One member of a pair was always presented in phase 2A and the other in phase 2B, an assignment that was determined randomly.

*Phase 2A, Imagination under Placebo 1 (Vitamin C Supplement).* Phase 2A consisted of 40 trials. On each trial, a name of a vacation destination appeared for 6 s. The participants imagined themselves vacationing at that location next year and then rated how happy they would be (from 1 = unhappy to 6 = extremely happy) and rated how vivid the image was in their mind (from 1 = low to 6 = high). A fixation cross was then presented for 3 s.

*Phase 2B, Imagination under L-DOPA (100 mg) or Placebo 2 (Vitamin C Supplement; Different Color and Size from Placebo 1).* Procedure was as in phase 2A.

*Ratings for Phase 2A and Phase 2B.* Reaction times decreased in phase 2B compared to phase 2A in both groups (see Supplemental Results), most likely as a result of practice. Absolute ratings of estimated pleasure and vividness did not differ between phase 2B and phase 2A in either group for either subsequently selected stimuli or rejected stimuli. Note that during these imagination sessions, L-DOPA stimuli and placebo stimuli were imagined and rated in separate sessions (phase 2A and phase 2B, respectively). Because participants may have used an absolute evaluation scale differently on phase 2A and phase 2B (i.e., the most pleasurable stimuli would be rated 6 in both conditions even if the most pleasurable stimuli under L-DOPA were more pleasurable than under placebo), we cannot reliably compare these ratings. In contrast, during phase 1 and phase 4, all stimuli were rated intermixed at the same time. Thus, those ratings, reported in the main text, can be reliably compared for the different types of stimuli.

#### Day Two

*Phase 3, Choice.* On each trial, two names of vacation destinations (see “Pair Construction” above) appeared on screen side by side for 4 s. The word “choose” then appeared above the two options for an additional 2 s, and participants indicated which location they would prefer to vacation at next year by pressing one of two buttons on the keypad. After the participants made a response, a star appeared next to the chosen location. Finally, a fixation cross was presented for 3 s. Choices were hypothetical.

*Phase 4, Rating 2.* Procedure was as in phase 1.

*Additional Rating Questionnaires.* Following phase 4, participants were asked to rate all stimuli on four scales (see [7]): previous visits, familiarity, vividness, and arousal. Three participants in each group failed to fill in these questionnaires. No group × condition interaction was found for any of these scores for either selected stimuli or rejected stimuli.

## Supplemental Data

Document S1. Two Tables, Supplemental Results, and Supplemental Experimental Procedures
