# Supplementary material for: Dopamine Enhances Expectation of Pleasure in Humans
Source: Curr Biol. 2009 Dec 29;19(24):2077–80. doi: 10.1016/j.cub.2009.10.025 (PMC2801060; doi:10.1016/j.cub.2009.10.025)
Supplement: Document S1. Two Tables, Supplemental Results, and Supplemental Experimental Procedures [file mmc1.pdf]

Current Biology, Volume *19*

Supplemental Data

Dopamine Enhances Expectation of Pleasure in Humans

Tali Sharot, Tamara Shiner, Annemarie C. Brown, Judy Fan, and Raymond J. Dolan

Table S1. Subjective State Questionnaire

	Experimental Group		Order Control Group	
**Subjective State Questionnaire completed at Phase 2A and Phase 2B**	**Placebo1**	**L-DOPA**	**Placebo1**	**Placebo2**
Alert (1) - Drowsy (6)	3.14	3.37	3.41	2.96
Calm(1) - Excited (6)	2.29	2.25	2.31	2.58
Strong(1) - Feeble (6) **	**2.62**	**3.11**	3.13	3.06
Muzzy (1) -Clear Headed (6)	3.51	3.51	3.51	3.75
Well Coordinated (1) - Clumsy (6)	2.48	2.07	2.82	2.75
Lethargic (1) - Energetic (6)	3.07	3.18	**2.96**	**3.44**
Contented (1) - Discontented (6)	2.03	2.11	2.41	2.58
Troubled (1) - Tranquil (6)	4.59	4.44	4.51	4.17
Mentally slow (1) - Quick witted (6) **	3.37	3.22	**3.17**	**3.62**
Tense (1) - Relaxed (6)	4.77	4.66	4.27	4.20
Attentive (1) - Dreamy (6)	3.66	3.51	**3.62**	**3.00**
Incompetent (1) - Proficient (6)	3.96	3.81	3.75	3.75
Happy (1) - Sad (6)	2.03	2.18	2.37	2.48
Antagonistic (1) - Friendly (6)	**4.62**	**4.14**	4.31	4.37
Interested (1) - Bored (6) **	**2.44**	**2.96**	2.96	2.96
Withdrawn (1) - Sociable (6)	4.00	3.81	3.72	3.58

Bold font indicates significant difference within group $p < 0.05$.
** Significant Group X Condition interaction $p < 0.05$
Three participants in the control group and two in the experiment group failed to fill questionnaires according to guidelines. Their data could not be included in analysis incorporating subjective state scores.
Questionnaire was adapted from [1].

Note: the subjective state questionnaires revealed that L-DOPA had a negative effect on subjective feeling of interest, physical strength, and mental slowness. This is most likely due to L-DOPA's known side effects, which include low blood pressure, nausea, and sleepiness [2]. Consequently, these scores were included as covariates in all subsequent analyses, which did not change the pattern of results. Note that L-DOPA did not alter the subjective experience of happiness, suggesting that L-DOPA does not simply induce a feeling of pleasure.

Table S2. Raw scores (before Mean Correction, Uncorrected for Subjective State)

		Pre-choice		Post-choice	
		Selected	Rejected	Selected	Rejected
Experiment Group	L-dopa	4.40	4.40	4.70	4.20
	Placebo	4.40	4.40	4.50	4.20
Control Group	Placebo1	4.20	4.20	4.40	3.90
	Placebo2	4.20	4.20	4.40	4.00

Supplemental Results

Consistent with previous findings [2, 3], there was a main effect of choice ($p < 0.0001$), characterized by an increase in mean-corrected hedonic ratings after the decision stage for selected stimuli, and a decrease for rejected stimuli. This effect is usually accounted for by cognitive dissonance theory [4]. However, alternative accounts have been suggested [2, 5]. An interaction between group and choice ($p < 0.05$) revealed that this difference (i.e., the post-choice increase in ratings for selected stimuli and the post-choice decrease in ratings for rejected stimuli) was more pronounced in the control group. This is because while choice was the *only* factor affecting post-choice rating change in the control group, pharmacological manipulation was an additional factor affecting rating change in the experiment group (as shown by the group x condition interaction reported in the main text). This therefore slightly weakened the independent observable effect of choice on rating change when averaging *over* L-DOPA and Placebo. There was also a non-significant trend for a main effect of condition ($p < 0.1$), such that ratings of stimuli imagined second were more likely to increase than stimuli imagined first.

As expected, an (ANOVA) (Group: *experiment/order-control* X Condition: *Placebo1/L-DOPA-or-Placebo2* X Choice: *selected/rejected*) on raw scores (not mean-corrected) revealed the same findings as the analysis on mean-corrected scores: an interaction between group and condition ($p < 0.05$), an interaction between group and choice ($p < 0.05$), and a main effect of choice ($p < 0.0001$).

Reaction times in both groups decreased in Phase 2B (L-dopa experiment group mean = 1.6s / placebo 2 control group mean = 1.4s) compared to Phase 2A (placebo1 experiment group mean = 1.9s / placebo1 control group mean = 1.8s) (both $p < 0.0001$), most likely due to practice. Importantly, there was no group (experiment/ control) by condition (Placebo1/L-DOPA-or-Placebo2) interaction $p > 4.3$.

Supplemental Experimental Procedures

Instructions Presented during Phase 1 and 4 (Rating)

“On each trial a name of a vacation destination will be presented on screen for 2sc.
Then you will rate how you expect to feel if you were to vacation at that destination next year.
The scale is:

1(unhappy) 2(a bit unhappy) 3(Neutral) 4(Happy) 5(VERY Happy) 6(EXTREMELY Happy)

You have 2sc to respond by pressing the appropriate button (1-6) when the scale comes up. Then a fixation cross will appear”.

Instructions Presented during Phase 3 (Choice)

“On each trial two names of vacation destinations will be presented on screen.
When the word CHOOSE comes up indicate which of the two you would rather vacation at next year.
If you would like to choose the one on the LEFT press 1.
If you would like to choose the one on the RIGHT press 2.
A star will appear next to the one you had chosen”.

Supplemental References

1. A. J. Bond, M. H. Lader, Br. J Med. Psychol. 47, 211 (1974). *British national formulary: 57,*1st ed. (Pharmaceutical Press, 2009).
2. T. Sharot, B. De Martino, R. J. Dolan, How Choice Reveals and Shapes Expected Hedonic Reaction. *The Journal of Neuroscience* 29, 3760.
3. J.W Brehm, Post-decision changes in the desirability of choice alternatives. *Journal of Abnormal and Social Psychology* 52, 389 (1956).
4. L. Festinger, *A theory of cognitive dissonance* (Stanford University Press, Stanford, CA. 1957).
5. D.J. Bem, Self-perception: An alternative interpretation of cognitive dissonance phenomena. *Psychological Review* 74, 183(1967).
